# The type of forest edge shapes snail assemblages at forest–pasture transitions

**DOI:** 10.1038/s41598-023-43758-8

**Published:** 2023-10-05

**Authors:** Dénes Schmera, Cristina Boschi, Bruno Baur

**Affiliations:** 1 Balaton Limnological Research Institute, Klebelsberg K. u. 3, 8237 Tihany, Hungary; 2 Department of Environmental Sciences, University of Basel, Bernoullistrasse 30, 4056 Basel, Switzerland; 3 Gränichen, Switzerland

**Keywords:** Ecology, Biodiversity, Community ecology, Conservation biology

## Abstract

Semi-natural, nutrient-poor calcareous grasslands are local biodiversity hotspots that are increasingly threatened by land use intensification, abandonment, or indirect effects from adjacent habitats. The habitat quality of these grasslands is often influenced by neighbouring forests or intensively managed agricultural land. For example, shrubs encroaching on grassland reduce the sensitive habitat, but at the same time represent a new habitat type (transition zone at gradual forest edge). We investigated the effects of gradual and abrupt forest edges on the species richness, abundance, species composition, functional diversity and number of species of conservation importance (red-listed species) of land snail assemblages at forest–pasture transitions in the Jura Mountains, Switzerland. Forest edge type influenced the snail assemblages in different ways. Transition zones at abrupt forest edges had a higher species richness and more snail individuals than transition zones at gradual forest edges. Transition zones also differed in land snail species composition. At gradual forest edges, the transition zones contained some openland snail species, while those at abrupt forest edges had a similar species composition to the forest interior. Functional diversity was significantly higher for snails in the forests and transition zones at both abrupt and gradual edges than in pastures. In contrast, pastures and transition zones at both abrupt and gradual edges had a significantly higher number of red-listed snail species. Based on our findings, we recommend the creation of gradual forest edges through regular forest management practices, rather than through shrub encroachment into pasture, which could reduce the size of the threatened habitat.

## Introduction

As human-made habitats, dry, nutrient-poor calcareous grasslands harbour numerous species including land snails whose primordial habitats (floodplains, peatlands, and rocky outcrops) have been largely destroyed in Western Europe^1^. A high conservation value is therefore attributed to these grasslands^2^. However, nutrient-poor calcareous grasslands are fragile because their maintenance depends on traditional farming techniques^3^. During the twentieth century, increasing pressure for higher production at low costs has led to either an intensification of grassland use (increased stocking rate and/or increased use of fertilizer) or to abandonment^4,5^. Both processes lead to a reduction of the area of semi-natural grassland. In the Jura Mountains in Switzerland, the remaining fragments of semi-natural grassland are frequently surrounded by forest or intensively cultivated agricultural land and thus isolated^6^. In this region, abrupt forest edges are frequently human-made with the intention to maintain the grassland for agricultural use. However, when grazing ceases, the forest expands into the grassland, which is a natural process of succession resulting in gradual forest edges and reduction of the grassland area.

The transition zone between forest and adjacent openland, also called ecotone, may represent habitat features of both ecosystems and may have a high structural diversity. As a result, the transition is inhabited by both forest and openland species, and may also harbour characteristic “ecotonal species”^7,8^. The width of the transition zone depends on the contrast between ecological features of the two adjacent ecosystems, which include environmental, structural and compositional variables^9,10^. At forest–grassland boundaries, the width of the transition zone depends on forest type, habitat age and forest edge type^10,11^.

Two basic forest edge types can be distinguished. Abrupt forest edges are often maintained at their point of creation, and are characterized by the overhanging canopy of branches growing into open land, and a dense understorey between large tree trunks^9^. In contrast, gradual forest edges are characterized by dense vegetation that gradually decreases in height toward the open space^9^. Gradual forest edges often result from a lack of maintenance; saplings, young trees and shrubs grow on the originally open area^9,10^. Therefore, gradual forest edges provide a transition zone with less sharp changes in abiotic factors and higher structural habitat complexity than abrupt forest edges^12^. Understanding the processes that change community composition along forest–grassland gradients is therefore of paramount importance for the preservation, maintenance and management of biodiversity in forests and grasslands.

Numerous studies have examined the abundance and/or taxonomical diversity (species richness or species diversity) of different organisms inhabiting forest edges (e.g., in plants^13,14^, arthropods^15,16^, birds^17^, and mammals^18^). Abundance and taxonomical diversity, however, provide little information about the mechanisms that shape communities, although such information is essential for biodiversity research^19^. Differences (or similarities) in species traits are key factors in evaluating species assemblages^19,20^. Various environmental characteristics can act as filters in the transition zone: species with suitable traits and tolerance limits can persist, while species lacking these traits or tolerance limits are filtered out^21,22^. Functionally similar species are likely to utilize the same resources^23^. Thus, functional information can be used to quantify differences between organisms in a community.

The influence of forest edge zone on the structuring of animal communities was mainly investigated in animals with good mobility (e.g., in birds^17^, mammals^24^, arthropods^25–27^). The aim of our study was to investigate whether the type of forest edge affects the pure grassland area, an increasingly rare habitat type. We investigated how transition zones at abrupt and gradual forest edges act as filters in shaping land snail assemblages. Land snails are an excellent model group for assessing small-scaled changes in their environment because of their limited mobility^28–30^. Terrestrial gastropods are also important decomposers^31^ and are especially sensitive to land-use changes^32–34^. Many snail species exhibit a high degree of habitat specialization with significant changes in richness and abundance over a few metres^35^.

We assessed the abundance, species richness and composition, and functional diversity of land snails along transect lines running from forest interiors through gradual or abrupt forest edges to nutrient-poor pastures in the Swiss Jura Mountains. In particular, we addressed the following questions: (1) Do species richness and snail abundance differ between the three habitat types (forest interior, transition zone and pasture) and between the transition zones of abrupt and gradual forest edges? (2) Do the three habitat types harbour distinct snail assemblages? (3) Does the snail assemblage in the transition zones of abrupt forest edges differ from that at gradual forest edges? (4) Can the three habitat types be characterized by indicator species and by snail species with special habitat requirements? (5) Does functional diversity of snails differ between the three habitat types and between the transition zones of abrupt and gradual forest edges? (6) Do the three habitat types (and the two forest edge types) differ in the number of species with conservation value (red-listed species)?

## Results

### Characteristics of the three habitat types

PCA indicated that forests, transition zones, and pastures show some degree of separation regarding the plot-level environmental characteristics (Fig. S1). Plots in the forest interior were characterized by the amount of canopy closure, deadwood, litter layer thickness, and leaf litter cover, while those in pasture were characterized by grass, old grass and herbs. Differences between forest and pasture explained 48.6% of the variability in the environmental data at abrupt forest edges and 48.8% at gradual edges.

Considering the two forest edge types, the transition zones of abrupt and gradual forest edges differed in habitat characteristics. PCA biplots demonstrate that habitat characteristics of forest and pasture partly overlapped in the transition zones of abrupt forest edges, with a large influence of forest characteristics (Fig. S1). In contrast, the transition zones of gradual forest edges can be viewed as a smooth changeover from grassland to forest (Fig. S1).

### Snail species richness and abundance

**Figure 1:**
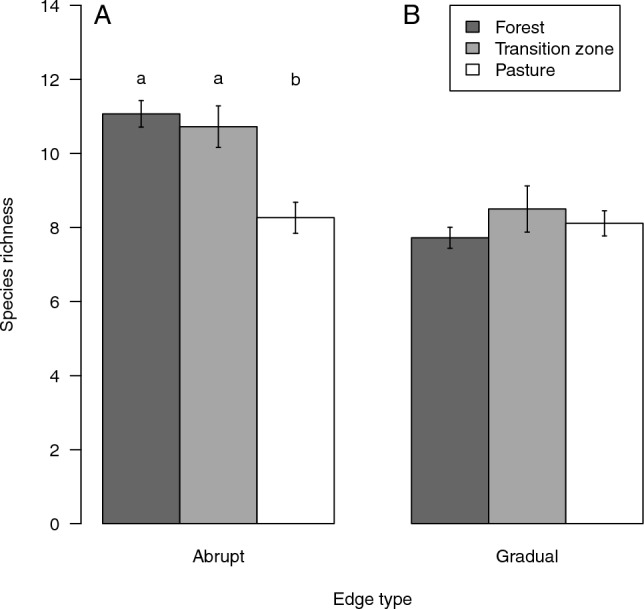
Species richness of snail assemblages in forests (dark grey), transition zones (light grey), and pastures (white) across abrupt (**A**) and gradual (**B**) forest edges. Bars show mean values (forests, n = 72; transition zone, n = 36; pasture, n = 72) and whiskers standard errors. Different letters indicate significant differences by Tukey test.

At abrupt forest edges, the species richness of snail assemblages differed between forest interior, transition zone and pasture (ANOVA: F_2,177_ = 14.259, *p* < 0.001; Fig. 1). In contrast, at gradual forest edges, snail species richness did not differ among forest, transition zone and pasture (ANOVA: F_2,177_ = 0.904, *p* = 0.407; Fig. 1). Our results also showed that pastures at abrupt forest edges harboured on average a lower number of snail species than forest interiors and the transition zones (Fig. 1). Considering the two edge types, the transition zones of abrupt forest edges showed a higher snail species richness than the transition zones of gradual forest edges (t-test, t = 2.656, df = 69.251, *p* = 0.009).

Individual-based rarefaction curves showed that the transition zones had greater rarefied species richness than forest interior and pastures at both abrupt and gradual forest edges (Fig. S2). In addition, rarefaction curves indicated that the transition zones at gradual forest edges had slightly—but not significantly—greater rarefied species richness than transition zones at abrupt forest edges (Fig. S3).

**Figure 2:**
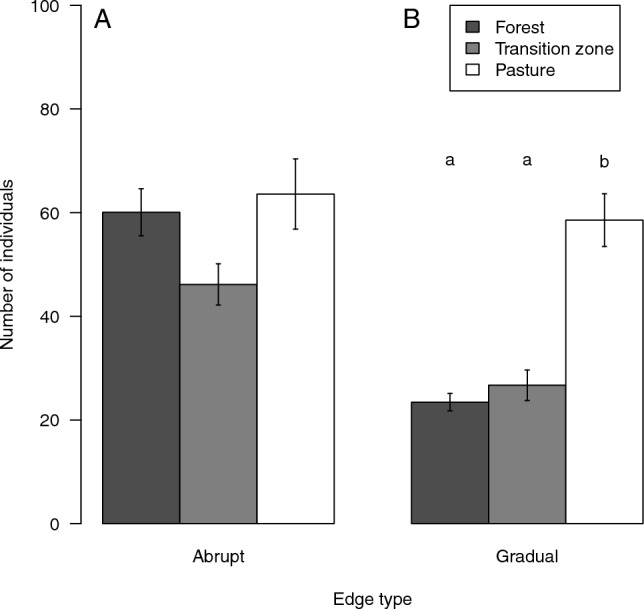
Abundance of snails in forests (dark grey), transition zones (light grey), and pastures (white) across abrupt (**A**) and gradual (**B**) forest edges. Bars show mean values (forests, n = 72; transition zone, n = 36; pasture, n = 72) and whiskers standard errors. Different letters indicate significant differences by Tukey test.

Snail abundance (number of individuals collected per plot) at abrupt forest edges did not differ among forest interior, transition zone and pasture (ANOVA: F_2,177_ = 0.652, *p* = 0.523; Fig. 2). At gradual forest edges, snail abundance differed among the three habitat types (ANOVA: F_2,177_ = 17.264, *p* < 0.001; Fig. 2). Snail abundance was higher in pastures than in the transition zones and forest interior at gradual edges (Fig. 2). Considering the two edge types, the transition zones of abrupt forest edges hosted more individuals than the transition zones of gradual forest edges (t-test, t = 4.063, df = 66.409, *p* < 0.001).

### Snail species composition

**Figure 3:**
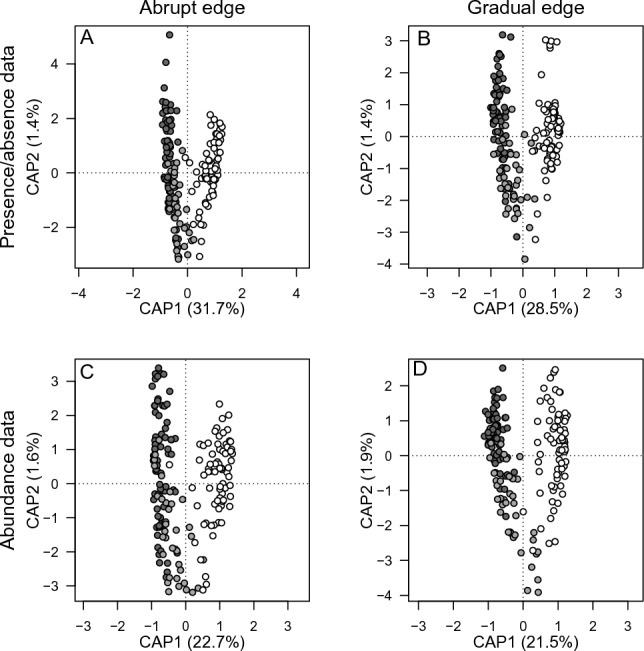
Ordination plots of constrained analysis of principal coordinates (CAP) of the snail assemblages. The plots show four different analyses: abrupt forest edge (**A**) and gradual forest edge (**B**), both based on presence/absence data, and abrupt forest edge (**C**) and gradual forest edge (**D**), both based on abundance data. Dark grey symbols indicate forest, light grey symbols transition zone, and white symbols pasture assemblages.

Constrained analyses of principal coordinates showed that the point clouds are well separated from forests, transition zones and pastures (Fig. 3). This suggests that the composition of snail assemblages of the three habitat types differed at both abrupt and gradual forest edges (Table 1, Fig. 3). Each analysis showed that the first constrained axis (CAP1) explained a higher amount of variation (ranging from 21.5 to 31.7%) than the second constrained axis (CAP2; ranging from 1.4 to 1.9%). This suggests that the species composition follows a single major gradient from the forest snail assemblage to the pasture snail assemblage (Fig. 3). At both abrupt and gradual forest edges, assemblages in the transition zone showed higher similarity to the forest assemblage than to the pasture assemblage (Fig. 3). Constrained analysis of principal coordinates also showed that the species composition of the snail assemblages differed between the transition zones of abrupt and gradual forest edges (presence/absence data [Sørensen distance]: ANOVA, F = 3.690, *p* = 0.001; abundance data [Bray–Curtis distance]: F = 4.796, df = 1,701, *p* = 0.001 for each case).

**Table 1:** Results of constrained analyses of principal coordinates examining whether abrupt and gradual forest edges differ in species assemblages of land snails (presence/absence and abundance data) in the forests, transition zones and pastures (df = 2,177 for each case).

	Abrupt forest edges	Gradual forest edges
Presence/absence data (Sørensen distance)	F = 43.829, *p* = 0.001	F = 37.890, *p* = 0.001
Abundance data (Bray–Curtis distance)	F = 28.355, *p* = 0.001	F = 28.886, *p* = 0.001

### Indicator species

At abrupt forest edges, indicator species analyses identified 14 indicator species for the forest interior, five for the transition zone, and eleven for the pasture (Table S1). At gradual forest edges, the corresponding numbers were nine, five and twelve indicator species (Table S1). Independently from the type of forest edge, indicators for forest interior were mainly species with a preference for forest, while indicators for pasture were typical openland species. However, a few ubiquitous indicator species occurred in either habitat type (Table S1).

Comparing the two forest edge types, the transition zones of abrupt edges harboured three indicator species with a preference for forest and two ubiquitous indicator species (Table S1). In contrast, the transition zones of gradual edges harboured three indicator species with a preference for forest, one with a preference for openland, and one ubiquitous indicator species (Table S1). Only the transition zones of gradual forest edges showed indicator species with a preference for openland. There was a relationship between habitat type (forest, transition zone and pasture), in which the snail species were recorded, and the habitat preference of indicator species (forest, openland and ubiquitous species) both at abrupt forest edges (Chi-squared = 26.626, df = 4, *p* < 0.001) and gradual forest edges (Chi-squared = 18.65, df = 4, *p* < 0.001).

### Habitat specificity

**Figure 4:**
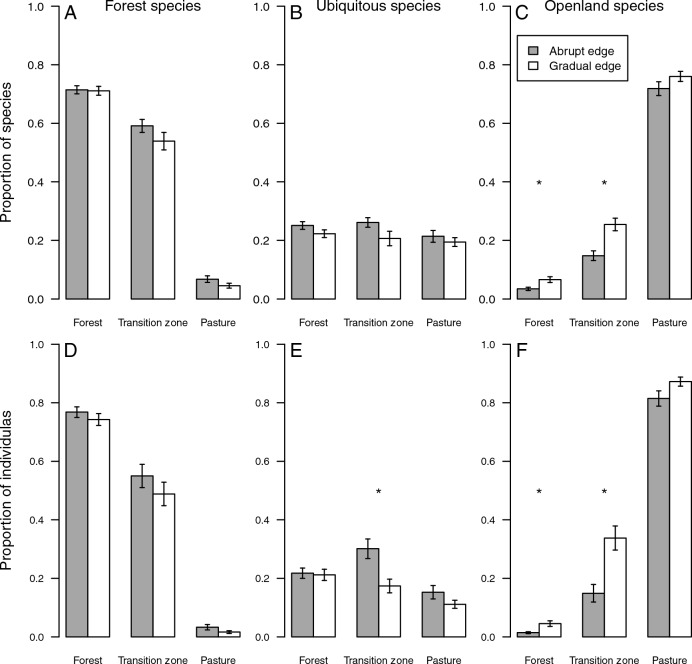
The proportion of typical forest (**A**,**D**), ubiquitous (**B**,**E**), and typical openland (**C**,**F**) snail species (**A**–**C**) as well as the respective number of individuals (**D**–**F**) that were recorded in the forest interior, transition zone and pasture (horizontal axis) at abrupt (grey) and gradual (white) forest edges. Bars show mean values (forests, n = 72; transition zone, n = 36; pasture, n = 72) and whiskers standard errors. Asterisk indicates significant differences by t-test.

The forest interior was dominated by typical forest species, while pastures by openland species (Fig. 4). Typical forest species were also frequently found in the transition zones at both abrupt and gradual forest edges (Fig. 4). In addition, the transition zones of abrupt forest edges contained several ubiquitous species, in contrast to the transition zones of gradual forest edges, which contained some openland species (Fig. 4). The proportions of openland species and openland individuals were higher in the forest interior and in the transition zones of gradual forest edges than in the corresponding habitats at the abrupt edges. Moreover, the transition zones of abrupt forest edges contained a higher proportion of individuals of ubiquitous species (Fig. 4).

### Functional diversity

**Figure 5:**
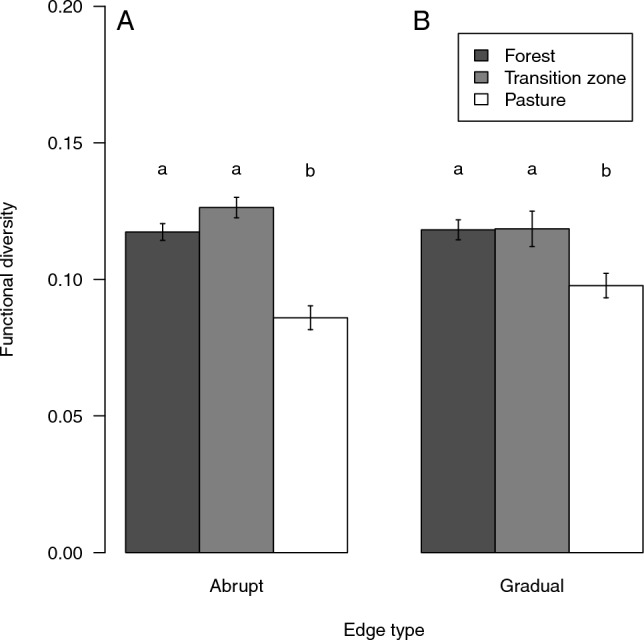
Functional diversity of snail assemblages in forests (dark grey), transition zones (light grey), and pastures (white) across abrupt (**A**) and gradual (**B**) forest edges. Bars show mean values (forests, n = 72; transition zone, n = 36; pasture, n = 72) and whiskers standard errors. Different letters indicate significant differences by Tukey test.

Functional diversity was significantly higher in snails in forests and in transition zones at both abrupt and gradual edges than on pastures (Fig. 5). The transition zones of abrupt and gradual forest edges did not differ in functional diversity in snails (t-test, t = 1.045, df = 56.087, *p* > 0.301).

### Species of conservation importance

Transition zones and pastures harboured similar numbers of snail species of conservation importance (Fig. S4). In contrast, the forest interior had a significantly lower number of such species than both transition zones and pastures (Fig. S4). The transition zones of abrupt and gradual forest edges did not differ in number of snail species of conservation importance (t-test, t = − 0.206, df = 64.096, *p* = 0.838).

**Figure 6:**
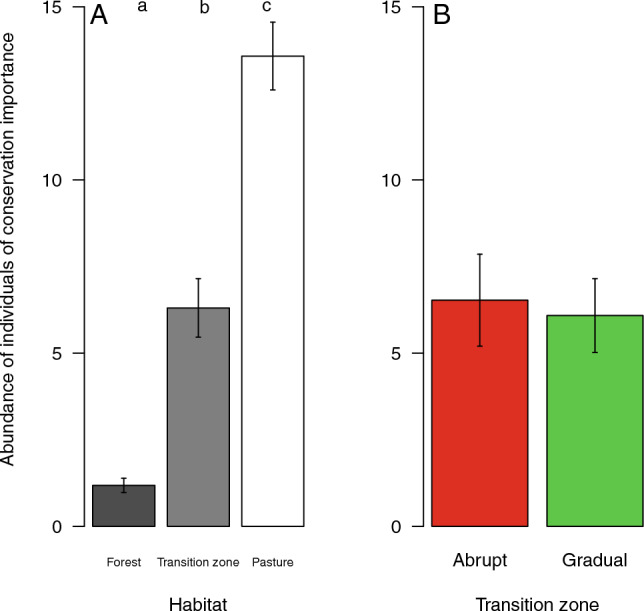
Number of snail species of conservation importance in forests (dark grey), transition zones (light grey), and pastures (white; **A**), and in the transition zones at abrupt and gradual forest edges (**B**). Bars show mean values (forests, n = 72; transition zone, n = 36; pasture, n = 72) and whiskers standard errors. Different letters indicate significant differences by Tukey test.

The three habitat types differed in abundance of snail individuals of conservation importance (Fig. 6). Pastures harboured a significantly larger number of individuals of conservation importance than transition zones and forests (Fig. 6). Transition zones also had more such individuals than forests (Fig. 6). However, the transition zones of abrupt and gradual forest edges did not differ in number of individuals of conservation importance (t-test, t = 1.261, df = 66.85, *p* = 0.795).

## Discussion

Our study showed that the snail species assemblages on pastures were clearly distinct from those of the transition zones at both types of forest edge and the forest interior. In addition, gradual and abrupt forest edges influenced the snail assemblages in a different way.

### Differences in environmental characteristics

Shrubs and trees in the forest provide shade and protection against wind. Daily fluctuations of air humidity and temperature are reduced in both the forest and transition zones, while the maximum temperature is higher in openland such as pastures^36^. In our study, the two transition zones also differed in environmental characteristics. Our analyses of environmental characteristics revealed that the transition zones at gradual forest edges represent a smooth changeover from forest to grassland, in contrast to a sharp change at abrupt forest edges. Other studies showed that abiotic factors in close proximity to gradual forest edges with an open canopy differ from those at abrupt forest edges with a dense canopy^9^. Because solar radiation penetrates deeper and stronger into gradual forest edges, air and soil temperatures are higher and relative humidity is lower at gradual than at abrupt forest edges^37^. Furthermore, the microclimate is more variable and the decomposition rates of leaf litter and woody debris are higher at gradual than at abrupt forest edges^9^.

### Differences in species richness and abundance

Forest edges represent not only a change in abiotic factors but also a change in structural diversity^12^. In some groups of organisms there are species that are specialized to transition zones (e.g., in Neuroptera^38^). Therefore, transition zones typically exhibit greater species diversity and often greater abundance of species than neighbouring habitats^10^. For example, transition zones hosted a greater number of spider, carabid and bird species than adjacent habitats^26,39,40^. In our study, rarefied snail species richness was greater in the transition zones than in the forest interior and pastures at both abrupt and gradual forest edges (Fig. S2). However, considering the observed species richness, snail species richness was greater in forest interiors and in transition zones at abrupt forest edges than on pastures, but did not differ between the three habitat types at gradual forest edges. Snails do not seem to benefit from an increased structural diversity at the forest edge. In the snail assemblages examined, all species prefer to stay on the ground or on ground vegetation. There are no true tree snails in these assemblages. Importantly, however, the open canopy in the transition zones at gradual forest edges allowed for the occurrence of typical openland species (e.g. *Helicella itala*). In this way, the habitat of these species is increased.

Considering snail abundance, we found more individuals on pastures at gradual forest edges than in forest interiors and in transition zones. However, in transition zones at abrupt forest edges, the three habitat types did not differ in snail abundance. A possible explanation for this finding is that forests with gradual edges generally harboured fewer snail individuals than forests with abrupt edges, although the underlying cause is not known.

### Differences in species composition

The distinct snail assemblages in the pastures and forests can be explained by the habitat preferences of the snail species, as the analysis of the indicator species show. The snail communities on the pastures consisted mainly of thermophilic species that tolerate dry and warm conditions typical of nutrient-poor, calcareous grasslands^1,41^. Thus, the microclimatic conditions in the forest interior are unfavourable for these snail species. In contrast, mainly snail species with a preference for forest and—to a lower number—ubiquitous species were found in the forests. The community compositions of the pastures and forests thus largely reflect the innate habitat specialization of the snails^34,41^.

Of particular interest is the difference in species composition between the transition zones at abrupt forest edges and those at gradual edges. Forest species were mainly found in the transition zones at abrupt forest edges. In contrast, open-land and forest species were recorded in the transition zones at gradual forest edges. This is likely due to less shading from the relatively open canopy near gradual forest edges compared to the dense canopy at abrupt forest edges. Magnin and Tatoni^42^ suggested that a forest snail community can establish when tree cover exceeds a 50% threshold. Another explanation could be the history of the areas. If gradual forest edges are caused by encroachment of shrubs and trees into the pastures, then open-land snails were already there and might just persist.

### Functional diversity

Functional approaches incorporating species traits provide a mechanistic understanding of how species communities are shaped by the environment^43^. In our study, functional diversity was significantly higher in snails in forests and in transition zones at both abrupt and gradual edges than on pastures. Snail species that inhabit nutrient-poor dry grasslands are highly specialized for harsh environmental conditions^41^. Thus, the vast majority of openland snail species have similar traits^44,45^. As a result, the snail community on pastures has a relatively low functional diversity. In contrast, the snail community in forests consists of species with very different traits, resulting in a higher functional diversity. The similarly high functional diversity in the transition zones can be explained by the great similarity in the species similarity with that in the forest. The few openland species that occurred in the transition zones at gradual forest edges did not result in a difference in functional diversity between the transition zones of the two edge types. Few studies have examined effects that may affect functional diversity in snail communities. In a controlled experiment, small-scale fragmentation of nutrient-poor, dry grassland reduced the functional diversity of snails in fragments in the seventh (final) year of the study^44^. In a field survey in 35 domestic gardens along a rural–urban gradient, snail functional diversity increased with habitat richness of the garden, but was not affected by the degree of urbanization and garden size^46^.

### Implications for management of grassland-forest edges

Nutrient-poor dry calcareous grasslands are a highly threatened habitat that harbours numerous rare and endangered plant and invertebrate species^1,2,45,47,48^. Among gastropods, some thermophilic snail species are threatened^49^. In our study, pastures and transition zones at both forest edge types hosted more conservation-relevant snail species than forests. These specialized snail species are endangered by progressive succession^6^ and by intensive grazing by cattle and sheep^32,33^. Shrubs encroaching in the pasture reduce the habitat for the specialized open-land species. In later succession stages, the overgrown grassland is no longer suitable for these species^50^.

Gradual forest edges are a desirable goal in nature conservation actions because gradual transitions generally favour higher biodiversity^51^. In most semi-natural grasslands in the Swiss Jura mountains (and elsewhere), gradual forest edges develop through shrub encroachment on the pastures reducing the area of pure grassland. If gradual forest edges are desired, they should be created through forestry activities^12,52^ (cutting back trees and shrubs in the forest), and not by allowing shrub encroachment of pastures.

## Methods

### Study sites

The study was carried out at 12 forest–pasture transitions located in an area of 18 km × 36 km in the Jura Mountains in northwestern Switzerland (Table S2). The pastures are surrounded by extensively used mixed deciduous forests with the dominant tree species European beech (*Fagus sylvatica*) and sycamore (*Acer pseudoplantanus*). All 12 forest edges examined were south facing. Six of them were gradual with a shrub strip from 1.1 to 7.4 m wide (mean: 4.2 m; hereafter referred to as gradual forest edge; Table S2). These shrubs spread from the formerly abrupt forest edge and overgrow the pasture. The other six forest edges showed a sharp change from grassland vegetation to forest trees (hereafter referred to as abrupt forest edge).

Abrupt edges tend to have a linear structure, while gradual edges have a certain width^9^. Therefore the transition zone is usually considered to compare the two edge structures^10^. We investigated the transition zone (5 m wide) both in gradual and on abrupt forest edges. We considered the sampling plot at the forest edge (near outermost tree or shrub, 0 m) and the sampling plot 5 m inside the forest as belonging to the edge zone. The distance of 5 m roughly corresponds to the width of the transition zone measured at gradual forest edges in our study. Other studies on arthropod diversity at forest edges in Europe considered the transition zone to be 5 m^25,26^.

**Figure 7:**
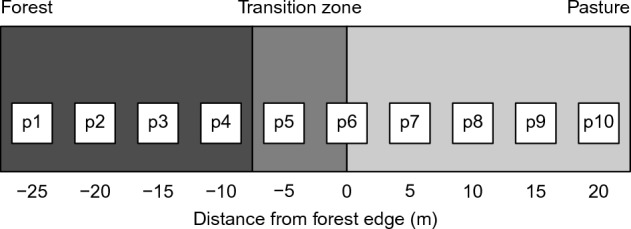
The sampling design with ten plots (p1 to p10) arranged in a row and running from forest through transition zone to pasture.

At each forest edge we established three parallel transect lines of sampling plots (measuring 1 m × 1 m) at a distance of 20 m. Transect lines were placed perpendicular to the forest edge, running from the forest interior (25 m) through the transition zone (5 m wide) at the forest edge into the grassland (20 m). We placed 10 sampling plots at 5 m intervals along each transect line. Four plots were set up in the forest interior, two in the transition zone (one exactly at the forest edge, i.e. close to the first trunk of bush or tree), and four plots in the pasture (Fig. 7). The length of the transect line was determined by the width of the extensively managed pastures. In the Swiss Jura Mountains, most extensive pastures are narrow strips (50–175 m wide) surrounded by forest, intensively managed agricultural land, and settlements^6^.

### Sampling methods

We collected land snails in the sampling plots between 19 April and 7 June 2006. We applied two methods to determine species richness and relative abundance of land snails. First, one person visually searched for live snails and empty shells in each sampling plot for 15 min. Second, we collected soil samples including dead plant material from randomly selected spots in each sampling plot (total 0.25 l of soil per plot). We dried the soil samples at 50 °C for 4 h. Samples were then passed through sieves with mesh sizes of 2, 1 and 0.2 mm and later examined under a binocular microscope. Snail shells were sorted out from the samples and identified according to Kerney et al.^53^. The nomenclature follows MolluscaBase^54^. We did not consider slugs because their activity depends heavily on weather conditions^55^, and the sampling methods used were not appropriate to determine slug abundance^56^.

### Site and plot characteristics

At the site level (n = 12), we recorded the following ecological variables: elevation (in metres above sea level, measured by a GPS receiver (Garmin, Geko 201, Romsey, U.K.) and checked against 1:25,000 topographical maps, geographical coordinates (measured with the GPS receiver), inclination (based on a trigonometric method, average of three measurements) and soil pH (average of three soil samples obtained from the pasture, forest edge and forest interior using the Hellige method; AVM Analyseverfahren, Freiburg, Germany; Table S2). Sites with abrupt and gradual forest edges did not differ in elevation (unpaired t-test, t = 1.075, n = 12, *p* = 0.31), inclination (t = 1.440, n = 12, *p* = 0.19) and soil pH (t = -0.0401, n = 12, *p* = 0.97).

At the 1-m^2^ plot level (n = 360), we assessed the percentages of bare ground, area covered by herbs, grass, old grass, stones, dead wood and leaf litter (all estimated to the nearest 5%; Table S3). We also measured the thickness of the latter layer (in cm) and estimated the canopy closure (estimated to the nearest 5%; Table S3).

### Snail traits and functional diversity

In terms of habitat preference, each snail species was assigned to one of the following categories: openland (species found exclusively in open habitat), forest (species found mainly in forests) or ubiquitous (species found in multiple habitat types) following Kerney et al.^53^ and Falkner et al.^41^.

We used Rao's quadratic entropy^57^ as a measure of functional diversity. To calculate functional diversity we used morphological (body size and shell shape) and life-history traits (age at sexual maturity and longevity) of the species recorded in the plots. Body size is expressed as shell size (the larger of shell height or width). Age at sexual maturity had an ordinal scale (< 1 year; 1 year; > 1 year) as had longevity (< 1 year; 1–2 years; > 2 years). Data were retrieved from Bengtsson and Baur^58^, Baur^59^ and Falkner et al.^41^.

Information on threatened species was obtained from the Red List of Switzerland for Molluscs^49^. Species were considered as threatened if they were classified as critically endangered, endangered, vulnerable or nearly threatened.

### Statistical analyses

For both forest edge types, we assigned the plots to three zones: (1) forest interior (plots at − 25, − 20, − 15, and − 10 m from the forest edge); (2) transition zone (plots at distances of − 5 and 0 m); and (3) pasture (plots at 5, 10, 15, and 20 m from the forest edge). We used Principal Component Analysis (PCA^60^) to examine which plot-level environmental variables characterize forest interiors, transition zones, and pastures at both abrupt and gradual forest edges. We applied Analysis of Variance (ANOVA) to compare snail species richness and abundance in forests, transition zone and pastures for abrupt and gradual forest edges separately. Abundance of snails was log-transformed to better approximate normality.

Individual-based rarefaction controls for differences in abundance, allowing comparison of species richness from habitats differing in the number of individuals collected. We used individual-based rarefaction^61^ to compare species richness between forests, transition zones, and pastures as well as between transition zones at abrupt and gradual forest edges.

We used Constrained Analysis of Principal Coordinates (CAP^62^) to examine whether the species composition of snail assemblages differed between forests, transition zones, and pastures at abrupt and gradual forest edges separately. CAP is an ordination method that shows variation that can be explained by constraining variables. We considered habitat type as constraining variable. In the analyses, we used both presence/absence (Sørensen distance) and abundance (Bray–Curtis distance) data. We run ANOVA-like permutations (n = 999) to test for significant differences in assemblage composition between habitat types.

Indicator species analysis^63^ was used to identify separately indicator species for the three habitat types (forest, transition zone, and pasture) at abrupt and gradual forest edges separately. Statistical analyses were performed in R^64^ using the packages vegan^65^, labdsv^66^ and FD^67^.

### Supplementary Information

Supplementary Information.

## Data Availability

Source data on the number of individuals for each species in each plot can be provided on a reasonable request to the first author. All other data generated or analyzed for this study are included in this published article.
