# Supplementary material for: The type of forest edge shapes snail assemblages at forest–pasture transitions
Source: Sci Rep. 2023 Oct 5;13:16761. doi: 10.1038/s41598-023-43758-8 (PMC10556092; doi:10.1038/s41598-023-43758-8)
Supplement: Supplementary file 1 — Supplementary Information. [file 41598_2023_43758_MOESM1_ESM.docx]

**Table S1.** List of indicator species for forest interior, transition zones and pastures (rows) at abrupt and gradual forest edges (columns). The habitat preference of each indicator species is given along with its indicator value (in parentheses). Nomenclature follows MolluscaBase^52^.

|  | Abrupt forest edge | Gradual forest edge |
| --- | --- | --- |
| Forest | *Cochlostoma septemspirale* FOREST (0.564) *Discus rotundatus* FOREST (0.462)  *Helicodonta obvoluta* FOREST (0.451)  *Acanthinula aculeata* FOREST (0.451)  *Acicula lineata* FOREST (0.391)  *Aegopinella nitens* FOREST (0.369)  *Carychium tridentatum* UBIQUITOUS (0.357)  *Vitrea subrimata* FOREST (0.357)  *Aegopinella pura* FOREST (0.348)  *Cochlodina fimbriata* FOREST (0.257)  *Orcula dolium* FOREST (0.176)  *Vitrea crystallina* UBIQUITOUS (0.154)  *Euconulus fulvus* UBIQUITOUS (0.153)  *Isognomostoma isognomostomos* FOREST (0.084)  *Arianta arbustorum* UBIQUITOUS (0.069) | *Discus rotundatus* FOREST (0.458)  *Cochlostoma septemspirale* FOREST (0.392)  *Helicodonta obvoluta* FOREST (0.373)  *Carychium tridentatum* UBIQUITOUS (0.307)  *Cochlodina fimbriata* FOREST (0.228)  *Monachoides incarnatus* FOREST (0.214)  *Vitrea subrimata* FOREST (0.199)  *Acicula lineata* FOREST (0.141)  *Cochlodina laminata* FOREST (0.139) |
| Transition zone | *Punctum pygmaeum* UBIQUITOUS (0.360)  *Merdigera obscura* FOREST (0.318)  *Abida secale* UBIQUITOUS (0.278)  *Cochlodina laminata* FOREST (0.268)  *Helicigona lapicida* FOREST (0.170) | *Vitrina pellucida* OPENLAND (0.341)  *Acanthinula aculeata* FOREST (0.282)  *Merdigera obscura* FOREST (0.217)  *Aegopinella pura* FOREST (0.187)  *Arianta arbustorum* UBIQUITOUS (0.056) |
| Pasture | *Vertigo pygmaea* OPENLAND (0.869)  *Vallonia excentrica* OPENLAND (0.771)  *Helicella itala* OPENLAND (0.652)  *Vallonia costata* OPENLAND (0.649)  *Pupilla muscorum* OPENLAND (0.462)  *Truncatellina cylindrica* OPENLAND (0.445)  *Vallonia pulchella* OPENLAND (0.403)  *Cochlicopa lubricella* OPENLAND (0.361)  *Candidula unifasciata* OPENLAND (0.264)  *Cecilioides acicula* OPENLAND (0.221)  *Perpolita hammonis* UBIQUITOUS (0.207) | *Vertigo pygmaea* OPENLAND (0.922)  *Vallonia excentrica* OPENLAND (0.821)  *Vallonia costata* OPENLAND (0.667)  *Pupilla muscorum* OPENLAND (0.648)  *Helicella itala* OPENLAND (0.578)  *Truncatellina cylindrica* OPENLAND (0.565)  *Vallonia pulchella* OPENLAND (0.417)  *Cochlicopa lubricella* OPENLAND (0.411)  *Trochulus hispidus* UBIQUITOUS (0.269)  *Perpolita hammonis* UBIQUITOUS (0.177)  *Candidula unifasciata* OPENLAND (0.111)  *Clausilia rugosa parvula* UBIQUITOUS (0.097) |

**Table S2.** Locations of the forest edges examined with elevation, inclination and soil pH of the sites in the Jura Mountains, Switzerland: g = gradual forest edge, a = abrupt forest edge; soil pH represents the average of three soil samples obtained from the pasture, forest edge and forest interior.

Pasture Locality Type of Coordinates Elevation Inclination Soil pH

forest edge N / E (m a.s.l.) (^o^)

Dittingerweid Dittingen g 47.4462 / 7.4936 520 23.6 6.4

Nenzlingerweid Nenzlingen g 47.4488 / 7.5683 520 19.4 5.3

Hellberg Liesberg g 47.3996 / 7.4075 585 23.7 7.3

Räschberg Liesberg g 47.4129 / 7.4263 700 23.9 7.3

Obere Wengi Matzendorf g 47.3253 / 7.6222 930 27.1 7.0

Haute Abateuse Movelier g 47.4153 / 7.3264 810 23.3 7.2

La Combe Soyhières a 47.4013 / 7.3594 590 33.9 7.3

Liesbergweid Liesberg a 47.4066 / 7.4168 590 22.2 7.0

Chli Weid Bretzwil a 47.3849 / 7.6343 910 20.8 5.5

Lauchweid Langenbruck a 47.3767 / 7.7889 990 30.0 6.8

Ulmet Lauwil a 47.3810 / 7.6539 990 27.1 6.8

Blauenweid Blauen a 47.4583 / 7.5244 670 26.2 7.0

_______________________________________________________________________________________________________

**Table S3**. Environmental variables [mean (± SE)] assessed in plots in the three habitat types (forest, transition zone and pasture) at both abrupt and gradual forest edges.

|  | Abrupt forest edge | | | Gradual forest edge | | |
| --- | --- | --- | --- | --- | --- | --- |
| Environmental variables | Forest^1^ | Transition zone^2^ | Pasture^1^ | Forest^1^ | Transition zone^2^ | Pasture^1^ |
| Open ground (%) | 2.00 (±0.49) | 11.17 (±2.91) | 5.03 (±0.67) | 5.06 (±1.35) | 8.22 (±2.29) | 8.51 (±0.99) |
| Old grass (%) | 2.88 (±0.87) | 2.89 (±0.93) | 7.42 (±0.80) | 1.07 (±0.26) | 4.58 (±1.95) | 10.5 (±0.98) |
| Grass (%) | 4.46 (±1.01) | 7.11 (±2.11) | 37.76 (±1.86) | 3.00 (±0.81) | 4.64 (±1.10) | 49.21 (±1.89) |
| Herbs (%) | 3.77 (±0.36) | 7.61 (±1.52) | 38.46 (±1.98) | 4.71 (±0.63) | 11.28 (±2.52) | 25.99 (±1.82) |
| Stones (%) | 17.93 (±2.41) | 14.25 (±2.82) | 3.36 (±0.69) | 3.36 (±0.71) | 1.17 (±0.48) | 0.93 (±0.20) |
| Deadwood (%) | 7.97 (±0.77) | 4.78 (±0.59) | 0.49 (±0.13) | 7.49 (±1.02) | 4.28 (±0.86) | 0.31 (±0.11) |
| Leaf litter cover (%) | 55.08 (±3.22) | 41.75 (±5.72) | 7.19 (±1.59) | 65.37 (±2.94) | 54.44 (±3.92) | 2.14 (±0.75) |
| Litter layer (cm) | 3.24 (±0.22) | 2.19 (±0.27) | 0.17 (±0.07) | 3.37 (±0.21) | 2.50 (±0.21) | 0.02 (± 0.01) |
| Canopy closure (%) | 77.63 (±1.91) | 77.75 (±4.03) | 8.82 (±2.59) | 74.07 (±2.41) | 65.69 (±5.73) | 0.42 (±0.29) |

^1^ n = 72 plots

^2^ n = 36 plots


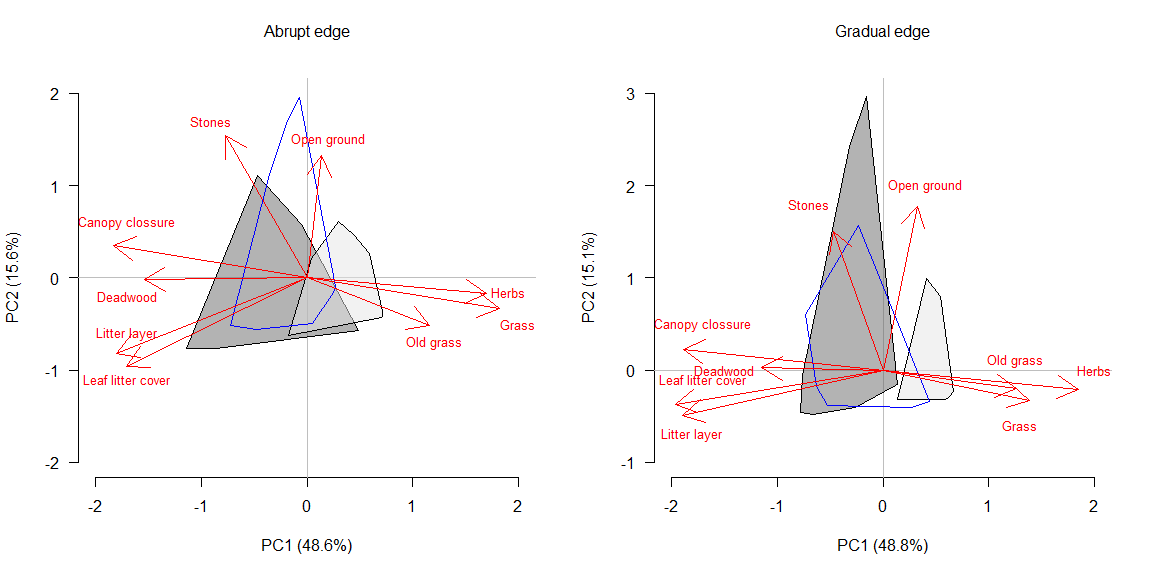


**Fig. S1.** Principal component analysis (PCA) biplot of quantified plot-level environmental characteristics in the forests, transition zones, and pastures across abrupt (left) and gradual (right) forest edges (red arrows). For clarity, plots are shown only as convex hulls representing forest (dark grey), pasture (light grey) and the transition zone (blue line).


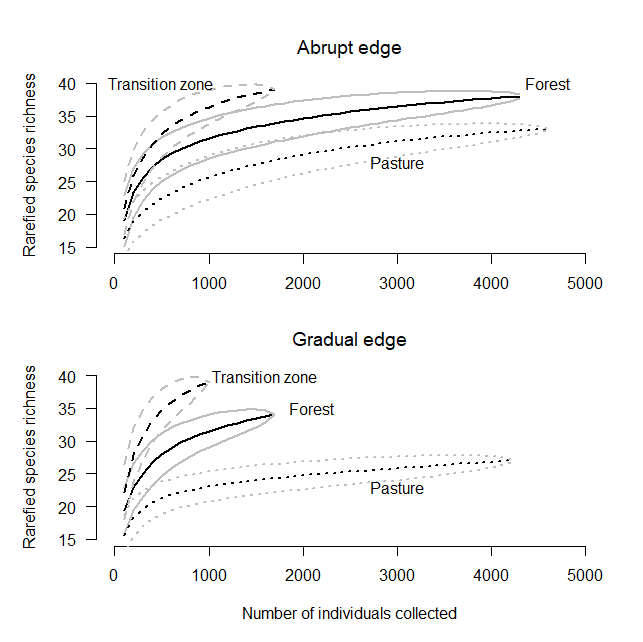


**Fig. S2.** Individual-based rarefaction curves of snail species richness for forests, transition zones and pastures at both abrupt and gradual forest edges, with 95% confidence intervals.


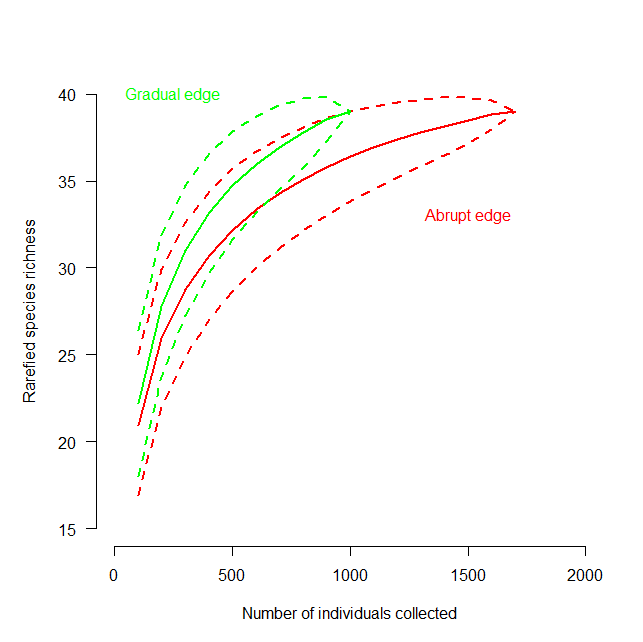
**Fig. S3.** Individual-based rarefaction curves of snail species richness for transition zones at abrupt (red) and gradual (green) forest edges, with 95% confidence intervals.


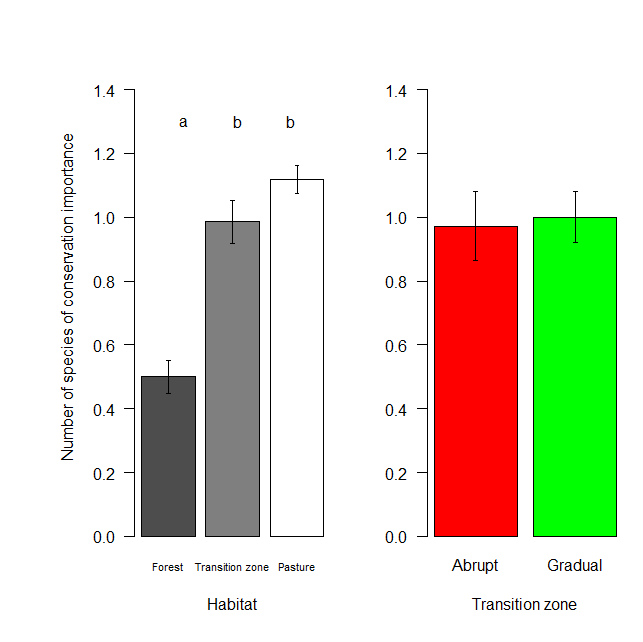


**Fig S4.** Number of snail species of conservation importance in forests (dark grey), transition zones (light grey), and pastures (white; left), and in the transition zones at gradual and gradual forest edges (right). Bars show mean values (forests, n = 72; transition zone, n = 36; pasture, n = 72) and whiskers standard errors. Different letters indicate significant differences by Tukey test.
